# Prävention und Gesundheitsförderung im und für das Alter stärken

**DOI:** 10.1007/s00391-023-02262-4

**Published:** 2023-12-13

**Authors:** Paul Gellert, Hermann Brandenburg, Annette Franke, Eva-Marie Kessler, Sonja Krupp, Johannes Pantel, Renate Schramek, Andreas Simm, Walter Swoboda, Susanne Wurm, Georg Fuellen

**Affiliations:** 1 Institut für Medizinische Soziologie und Rehabilitationswissenschaft, Charité – Universitätsmedizin Berlin, Charitéplatz 1, 10117 Berlin, Deutschland; 2 Friede Springer – Cardiovascular Prevention Center, Berlin, Deutschland; 3 Deutsches Zentrum für Psychische Gesundheit (DZPG), Standort Berlin/Potsdam, Deutschland; 4 Einstein Center for Population Diversity, Berlin, Deutschland; 5 Dekan, Pflegewissenschaftliche Fakultät, Vinzenz Pallotti University Vallendar, Vallendar, Deutschland; 6 Gesundheitswissenschaften, Soziale Gerontologie und Methoden und Konzepte der Sozialen Arbeit, Evangelische Hochschule Ludwigsburg, Ludwigsburg, Deutschland; 7 Department Psychologie, MSB Medical School Berlin, Berlin, Deutschland; 8 Forschungsgruppe Geriatrie Lübeck, Krankenhaus Rotes Kreuz Lübeck Geriatriezentrum, Lübeck, Deutschland; 9 Arbeitsbereich Altersmedizin mit Schwerpunkt Psychogeriatrie und klinische Gerontologie, Institut für Allgemeinmedizin, Goethe-Universität Frankfurt am Main, Frankfurt am Main, Deutschland; 10 Frankfurter Forum für interdisziplinäre Alternsforschung (FFIA), Goethe-Universität Frankfurt, Frankfurt am Main, Deutschland; 11 Gesundheitsdidaktik, Department of Community Health, Hochschule für Gesundheit Bochum, Bochum, Deutschland; 12 Universitätsklinik und Poliklinik für Herzchirurgie, Martin-Luther-Universität Halle-Wittenberg, Halle, Deutschland; 13 Praxis für Geriatrie und Innere Medizin, Würzburg, Deutschland; 14 Institut für Biomedizin des Alterns, Nürnberg, Deutschland; 15 Abteilung für Präventionsforschung und Sozialmedizin, Institut für Community Medicine, Universitätsmedizin Greifswald, Greifswald, Deutschland; 16 Institut für Biostatistik und Informatik in Medizin und Alternsforschung (IBIMA), Universitätsmedizin Rostock, Rostock, Deutschland; 17 Klinik und Poliklinik für Herzchirurgie, Martin Luther Universität Halle-Wittenberg, Ernst-Grube Straße 40, 06120 Halle (Saale), Deutschland

**Keywords:** Vorbeugung, Intervention, Interdisziplinarität, Multimorbidität, Prevention, Intervention, Interdisciplinarity, Multimorbidity

## Abstract

**Hintergrund:**

Prävention von Erkrankungen und Gesundheitsförderung im und für das Alter haben an Bedeutung gewonnen. Dennoch bedarf es mehr (nationaler) Forschung und Umsetzung in der Praxis, wie der internationale Vergleich zeigt.

**Ziel der Arbeit:**

Leitgedanken für Forschung und Praxis zu Prävention und Gesundheitsförderung im und für das Alter entwickeln.

**Material und Methoden:**

Im Rahmen eines iterativen Prozesses kamen Mitglieder der Deutschen Gesellschaft für Gerontologie und Geriatrie in Workshops und Symposien zusammen, um wesentliche Leitgedanken und Handlungsfelder zu Prävention und Gesundheitsförderung zu formulieren.

**Ergebnisse:**

Herausgearbeitet wurden: 1. Prävention und Gesundheitsförderung sind bis ins hohe Alter sinnvoll und möglich, 2. Prävention und Gesundheitsförderung für das Alter sollten früh beginnen, 3. Prävention und Gesundheitsförderung müssen die Diversität und Heterogenität der Lebenslagen alter Menschen aufgreifen, 4. Prävention und Gesundheitsförderung fördern und fordern Selbstbestimmung und Partizipation, 5. Prävention von Mehrfacherkrankungen gilt es, stärker in den Blick zu nehmen, 6. Prävention von Pflegebedürftigkeit und Prävention in der Pflege sind gleichrangig zu behandeln, 7. Prävention und Gesundheitsförderung müssen lebensweltlich und sektorenübergreifend gedacht werden, dabei sind v. a. Aspekte der sozialen Ungleichheit und eine Ressourcenorientierung mitzubeachten, 8. Prävention und Gesundheitsförderung und die Forschung dazu sind inter- und transdisziplinär und auf unterschiedlichen Ebenen anzulegen, von molekular bis gesellschaftlich.

**Diskussion:**

Die Leitgedanken spannen Schwerpunkte einer zukunftsgerichteten Alterns‑, Gesundheits- und Versorgungsforschung auf und öffnen Handlungsfelder, aber auch Grenzen dieses Zugangs für politische Entscheidungstragende, Forschende und Praktiker:innen.

**Zusatzmaterial online:**

Zusätzliche Informationen sind in der Online-Version dieses Artikels (10.1007/s00391-023-02262-4) enthalten.

## Abstract (en)

**Background:**

Disease prevention and health promotion in and for old age have become increasingly more important. Nevertheless, more (national) research and implementation in practice is needed, as the international comparison shows.

**Objective:**

To develop guiding principles for research and practice on prevention and health promotion in and for old age.

**Material and methods:**

As part of an iterative process, members of the German Society of Gerontology and Geriatrics came together in workshops and symposia to formulate key guiding principles and fields of action for prevention and health promotion.

**Results:**

The following were worked out: 1) prevention and health promotion are useful and possible up to oldest age, 2) prevention and health promotion for advanced age should start early, 3) prevention and health promotion must take into account the diversity and heterogeneity of the life situations of old people, 4) prevention and health promotion promote and demand self-determination and participation, 5) prevention of multiple illnesses must be given greater attention, 6) prevention of the need for long-term care and prevention in long-term care must be treated equally, 7) prevention and health promotion must be thought of in terms of life worlds and across sectors, paying particular attention to aspects of social inequality and a focus on resources, 8) prevention and health promotion and the related research must be interdisciplinary and transdisciplinary and be applied at different levels, from molecular to societal.

**Discussion:**

The guiding principles outline the focal points of future-oriented ageing, health and healthcare research and open up fields of action but also show the limits of this approach for political decision-makers, researchers and practitioners.

## Hintergrund

Die Gesellschaft des langen Lebens fordert Fokussierung hin zur Prävention von (Mehrfach‑)Erkrankungen und Folgeerkrankungen sowie auf die Gesundheitsförderung im Allgemeinen. Der demografische Wandel [25], der die Kranken- und Pflegesysteme zunehmend vor Herausforderungen stellt, drängt zu diesem Umdenken. Der medizinische Fortschritt und Trends wie Digitalisierung, größere Mobilität und Diversität bieten Chancen und Risiken für eine auf Prävention und Gesundheitsförderung ausgerichtete Gesellschaft. Das deutsche Gesundheitssystem ist angesichts des geringen Anteils von Prävention bei den allgemeinen Gesundheitsausgaben bislang zu wenig auf Prävention ausgerichtet, obwohl dieser Fokus auch ökonomisch nachhaltig wäre [21]. Mit dem Präventionsgesetz (PrävG) ist ein (wenn auch unzureichendes) Instrument vorhanden, um die Verhältnisprävention als Lebensweltansatz zu stärken [9]. So können durch das PrävG beispielsweise Gesundheitspotenziale pflegebedürftiger Menschen gezielt gefördert werden [9].

Befunde von BMBF-Forschungsverbünden für Prävention und Gesundheitsförderung für die Allgemeinbevölkerung konstatieren, dass Präventionsforschung angewandt und zwischen Forschung und Praxis aufgestellt sein sollte [30]. Der vorliegende Beitrag geht darüber hinaus, indem er die Präventionspotenziale und Themenfelder einer künftigen Forschung der Gesundheitsförderung und Prävention im Alter und für das Alter bestimmt.

## Methodisches Vorgehen

Das vorliegende Papier entstand in einem iterativen Prozess zwischen den Mitgliedern der Deutschen Gesellschaft für Gerontologie und Geriatrie (DGGG). Die DGGG steht für eine interdisziplinäre und breite Betrachtung der Prävention und Gesundheitsförderung im und für das Alter, die zentral in jeder zukünftigen Alternsforschung und Alterspolitik stehen muss. Mit ihren Sektionen und fachübergreifenden Ausschüssen (FA), die jeweils unterschiedliche Zugänge der Alternsforschung abbilden, stellt die DGGG die notwendige interdisziplinäre Bündelung von Expertise bereit. So kommen Perspektiven zu biologischen Prozessen des Alterns (Sektion I, Experimentelle Gerontologie), geriatrischer Medizin (Sektion II), der sozial- und verhaltenswissenschaftlichen Gerontologie (Sektion III) und der Sozialen Gerontologie und Altenarbeit (Sektion IV), hier auch zu Alter und Technik (FA), zur kritischen Gerontologie (FA) sowie zur Geragogik/Altersbildung (Arbeitskreis der Sektion IV) zusammen. Eine erste Version eines Papiers entstand als Entwurf durch die Erst- und Letztautoren (P.G., G.F.) am 19.03.2021 (Überarbeitungen: 23.05.2021 mit Rückmeldungen der Mitglieder des Präsidiums, der Vorstände und der FA und 22.07.2021 mit Rückmeldungen von Mitgliedern der DGGG, gefolgt von einer Diskussion auf den Mitgliederversammlungen der Sektionen III und IV [16.09.2021], Symposium Prävention aus allen vier Sektionen „Prävention, interdisziplinär“ [13.09.2022], Gründung AG Prävention und Gesundheitsförderung im Alter [13.09.2022], Zoom-Workshop 1 [20.01.2023; hier wurden in einem offenen Diskussionsformat die Punkte Format, Fokus, Publikationsorgan, Themenbereiche und Arbeitsweise besprochen], Zoom-Workshop 2 [12.06.2023; Diskussionspunkte: Vertiefung der Themenbereiche, Darstellung, Schlussfolgerungen, offene Punkte]). Die Dokumentation erfolgte ausschließlich im Protokoll, in weitergeleiteten kommentierbaren Arbeitsversionen und in einem geteilten Online-Dokument (ab 13.09.2022), an dem alle Beteiligten mitwirken konnten.

## Ergebnisse

**Figure 1:**
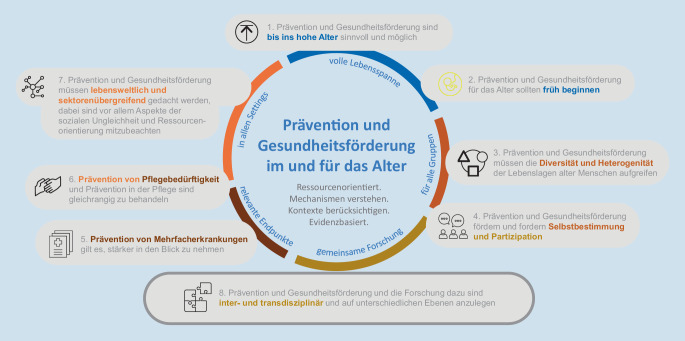

Folgende Leitgedanken wurden erarbeitet (Abb. 1):

### 1. Prävention und Gesundheitsförderung sind bis ins hohe Alter möglich.

Die Zunahme der weitgehend gesunden Lebensjahre ist aus demografischer und individueller Sicht auch zukünftig ein hoch relevanter Zielpunkt wirksamer Maßnahmen mit der Absicht der Prävention [2]. Die Wirksamkeit von Präventionsmaßnahmen wurde in der Vergangenheit vielfach nur in Gruppen relativ „junger“ und gesunder älterer Menschen untersucht. Obwohl es deutliche Forschungsdefizite für Präventionsmaßnahmen im hohen Alter gibt, existiert dennoch bereits eine Reihe robuster Befunde, die die Wirksamkeit von Prävention bis in das hohe Alter (z. B. [26]) und auch unter der Bedingung von Gebrechlichkeit („frailty“) (z. B. [27]) nahelegen. Eine moderne gerontologisch-geriatrische Präventionsforschung muss diese Datenbasis stärken und für verschiedene Gruppen älterer Menschen – v. a. für hochaltrige oder vulnerable Gruppen – ausbauen. Dies gilt für medikamentöse Präventionsansätze, aber besonders für nichtpharmakologische verhaltens- und verhältnispräventive Maßnahmen, etwa zur Prävention im psychischen, im physischen, im kognitiven oder im sozialen Bereich. Gerade in Kombination mit sozialen und digitalen/technischen Hilfen können dabei individuelle Potenziale und Bedarfe adressiert werden.

### 2. Prävention und Gesundheitsförderung für das Alter sollten früh beginnen.

Prävention und Gesundheitsförderung im Alter heißt auch, diese Ansätze im Lebenslauf mitzudenken; dies schließt frühe Lebensjahre und intergenerationale Prävention mit ein [18]. Kapazitäten können über den Lebenslauf aufgebaut werden, auch um kritische Lebensereignisse und -umstände bewältigen zu können, ergänzend zur Prävention im Alter. Gleichzeitig können sich jedoch auch Ungleichheiten und Defizite kumulativ über die Lebensspanne aufbauen [18]. Prävention im Lebenslauf ergänzt das Wissen, dass Prävention bis ins hohe Alter möglich ist, auf eine synergistische Weise [7]. Die Gesundheitsförderung und die Prävention von Erkrankungen, funktionellen Einbußen und der Beeinträchtigung von Alltagsaktivitäten im Alter durch lebensweltorientierte und lebensstilbezogene Interventionen umfasst auch den Aufbau von Ressourcen in der Kindheit oder im mittleren Erwachsenenalter, von denen Ältere profitieren können.

### 3. Prävention und Gesundheitsförderung müssen die Diversität und Heterogenität der Lebenslagen alter Menschen aufgreifen.

Prävention muss an die Unterschiedlichkeit der Lebenslagen und die Voraussetzungen von älteren Menschen und ihre Lebenswelten angepasst sein (Community-Bezug). Systematisch sollten die Diversitätskategorien, z. B. Alter, Geschlecht, Kulturzugehörigkeit, sexuelle Identität, Bildungsstand sowie deren Überschneidungen berücksichtigt werden. Nicht jeder ältere Mensch profitiert gleichermaßen von einer bestimmten Gesundheitsförderungs- oder Präventionsmaßnahme (oder ist empfänglich dafür). Präventionsforschung sollte daher im Blick haben, gesundheitliche Ungleichheiten durch entsprechende Maßnahmen zu verringern und auf gesundheitliche Chancengleichheit zu zielen, Community Health ist ein zentraler Zugang dafür [15]. Zu beachten gilt, dass auf Individualebene ansetzende Maßnahmen das Risiko bergen, sozioökonomische Ungleichheiten zu verstärken, weil ihre Effektivität an eine hohe Agency von Individuen gebunden ist [10].

### 4. Prävention und Gesundheitsförderung fördern und fordern Selbstbestimmung und Partizipation.

Die Ottawa-Charta der Weltgesundheitsorganisation bezeichnet Gesundheitsförderung als einen Prozess, der neben der Schaffung gesundheitsförderlicher Lebenswelten auch die Selbstbestimmung der Menschen stärkt. Prävention und Gesundheitsförderung sind entsprechend kein Selbstzweck, sondern gründen auf dem Recht auf Entwicklung des jeweils individuellen Gesundheitspotenzials eines jeden Menschen. Zugleich erfordern diese u. a. für Entscheidungen ein bestimmtes Maß an Gesundheitskompetenz und Partizipation. Daher zielt eine moderne Präventions- und Interventionsforschung auf die Entfaltung von Gesundheitspotenzialen der Menschen, auf edukative Begleitung und unterstützt die Ausbildung von Kompetenzen zur gesundheitsbezogenen Selbstbestimmung und Partizipation [12]. Hier sind strukturelle Überlegungen mitzudenken, welche Umwelten und Regulationen, Selbstbestimmung und Partizipation ermöglichen. *Empowerment *von älteren Menschen, auch und gerade angesichts von Unterstützungsbedarf, muss Ziel von Präventionsforschung sein und verstärkt als Endpunkt einer wirksamen Präventionsforschung neben rein klinischen Endpunkten stehen. Dies betrifft neben der Wirkung der Interventionen auch den Forschungsprozess selbst, der partizipativ zu gestalten ist. Partizipative Präventionsforschung zu Prävention und Gesundheitsförderung erlaubt es, die personen- und gruppenspezifische Nutzbarkeit und Relevanz zu adressieren.

Ein weiterer Punkt ist die kritische Auseinandersetzung mit Altersstereotypen und damit Verallgemeinerungen auf den „typischen alten Menschen“, was der großen Heterogenität in der Gruppe Älterer widerspricht. Eine moderne und inklusive Präventionsforschung trägt der hohen Vielfalt im Alter Rechnung und vermeidet, einseitig nur „die fitten Alten“ (Potenzialperspektive) zu adressieren oder Menschen als „zu alt für Prävention“ (Defizitorientierung) anzusehen. Auch hat die mittlerweile gesellschaftlich stark ausgeprägte Erwartungshaltung, im Alter „fit und aktiv“ zu sein, grundsätzlich ein hohes Potenzial für das Gelingen eines guten Alterns [11]. Allerdings muss darauf geachtet werden, dass es im Fall unzureichender Befolgung der Aktivitätsnorm durch alte Menschen – sei es wegen Unvermögen oder Unwillen – nicht zu Kritik oder gar Sanktionen oder Ausgrenzung kommt. Die kritische Reflexion gesellschaftlicher Altersbilder und -normen ist erforderlich, um stereotype Vorstellungen und Muster aufzubrechen und damit zu Gesundheit und Langlebigkeit älterer Menschen beizutragen [29].

### 5. Prävention von Mehrfacherkrankungen stärker in den Blick nehmen.

Präventionsmaßnahmen setzen meist spezifisch auf die Vermeidung einzelner Erkrankungen. Gerade im hohen Alter greift dieser Ansatz häufig zu kurz. Obwohl es zur Prävention von Multimorbidität eine noch unzureichende Evidenzlage gibt, sind psychosoziale, rehabilitative und verhaltensbezogene Faktoren, einschließlich der umfassenden Perspektive der sozialen Determinanten der Gesundheit, zentrale Stellschrauben [23]. Eine erkrankungsübergreifende Prävention im Alter kann heißen, besonders auf den Erhalt von Funktionsfähigkeit und Verhinderung von Gebrechlichkeit [1], Prävention von Multimorbidität [23], Erhalt einer guten subjektiven Gesundheit und Lebensqualität [24] oder die allgemeine Gesundheitsförderung zu fokussieren [16]. Dabei ist es auch wichtig, das Zusammenspiel und die Prävention von (eher) körperlichen und (eher) mentalen Erkrankungen zu beachten. Die Evaluierung von Präventionsmaßnahmen im Alter erfordert, dass Multimorbidität als Indikator für die Effektivität einer Intervention berücksichtigt wird [10].

### 6. Prävention von Pflegebedürftigkeit und Prävention in der Pflege.

Die Prävention des Auftretens und Fortschreitens von Pflegebedürftigkeit sollte zentrales Anliegen einer Präventionsstrategie sein. Sie umfasst ein breites Spektrum von Interventionen, einschließlich der (geriatrischen) Rehabilitation, der Beratung älterer Menschen im ambulanten Bereich [6], der ambulanten, mobilen und (teil-)stationären rehabilitativen Therapie im interdisziplinär arbeitenden Geriatrie-Team (ob § 109 oder § 111 SGB V zugeordnet) und der Prävention in Einrichtungen der Altenpflege (vgl. Präventionsgesetz 2015, Bundesrahmenempfehlungen der Nationalen Präventionskonferenz nach § 20d Abs. 3 SGB V) und kann selbst nach Eintritt von Pflegebedürftigkeit, z. B. über multidimensionale Bewegungsförderung, die verbliebenen Anteile der Selbstständigkeit länger erhalten und diese sogar ausbauen [14]. Breite Präventionsansätze schließen die Förderung besserer Ernährung und sozialer Kontakte ein und können zu mehr Lebensqualität und Gesundheit beitragen. Die COVID-19-Pandemie hat deutlich gezeigt, wie wichtig Prävention im Setting Pflegeheim ist – sowohl infektiologisch als auch im Sinne der größtmöglichen Reduktion unerwünschter Wirkungen der ergriffenen Schutzmaßnahmen [19]. Insgesamt muss der rehabilitativ-aktivierende Fokus der Pflegearbeit deutlicher ins Zentrum gerückt werden. (Für ein Modellprojekt in der stationären Langzeitpflege zur „rehabilitativen Altenpflege“: [4]). In der (teil-)stationären Pflege gilt es, durch geeignete und (auch) akademisch geschulte Pflegefachkräfte a) das pflegepräventive Potenzial durch ein Assessment diagnostisch zu erfassen, b) entsprechende Maßnahmen umzusetzen und c) eine Evaluation der Prozesse und erzielten Effekte systematisch vorzunehmen. Prävention muss künftig aber auch pflegebedürftige Menschen im ambulanten Umfeld und ihre Unterstützenden erreichen, wenn der Ruf „ambulant vor stationär“ Sinn ergeben soll. Dem steht in der Versorgungsrealität derzeit u. a. ein Mangel an präventiv wirksamen Angeboten (neben individueller Physio- und Ergotherapie auch geeignete Trainingsprogramme in der Häuslichkeit oder in Gruppen, z. B. Rehabilitationssport) entgegen. Ein Ausbau von aufsuchenden Angeboten, Transportmöglichkeiten und digitalen Angeboten (etwa per Videokonferenz) ist dringend notwendig.

### 7. Prävention und Gesundheitsförderung lebensweltlich und sektorenübergreifend denken, dabei sind v. a. Aspekte der sozialen Ungleichheit und Ressourcenorientierung mitzubeachten.

Für das generationenübergreifende gesundheitliche Wohlbefinden sind die lokalen Bedingungen wie gesundheitliche und pflegerische Infrastruktur (inkl. Netzwerkarbeit und Untersuchungs- und Beratungsangebote für Menschen in diversen Lebenslagen) sowie das Gesundheitsmonitoring von entscheidender Bedeutung [8]. Neben einer primär erkrankungsbezogenen Prävention ist es wichtig, gesundheitsförderliche und präventive Maßnahmen in bewährte Politikansätze wie *Health in All Policies* auf kommunaler Ebene oder *Age-friendly Cities and Communities *einzubetten und dabei individuelle Ressourcen und Lebenswelten zu beachten. Der Sachverständigenrat zur Begutachtung der Entwicklung im Gesundheitswesen des Bundes hat bereits 2009 in einem Gutachten Empfehlungen für gesunde, zukunftsfähige Metropolen formuliert: Koordination und Integration der Gesundheitsversorgung auf regionaler Ebene für alle Generationen. Dabei sind die rechtlichen Rahmenbedingungen im Sinne des in 2015 verabschiedeten Präventionsgesetzes für kommunale Gestaltungsmöglichkeiten bisher nicht ausgeschöpft [8]. Im Kern geht es darum, ganzheitlich ökonomisch und sektorenübergreifend zu planen und vernetzt zu denken, für Sektoren wie „stationäre und ambulante Versorgung“, „Pflege“, „Rehabilitation“, „Prävention und Gesundheitsförderung“ sowie „Stadtentwicklung“, „Energie“, „Verkehrsplanung“ und „Digitalisierung“. Es ist obligatorisch, dass nichtärztliche Professionen viel mehr, als dies bisher der Fall ist, als gleichberechtigte Akteur:innen (z. B. Psycholog:innen/Psychotherapeut:innen, Gerontolog:innen, Geragog:innen, Sozialarbeiter:innen, Ergotherapeut:innen, Physiotherapeut:innen, Therapeut:innen im Bereich künstlerischer Therapien) eingebunden werden, wie es in angloamerikanischen und skandinavischen Ländern der Fall ist. Auch findet derzeit ein gesundheitspolitischer Prozess der Dezentralisierung und Regionalisierung der Gesundheitsversorgung einschließlich Prävention und Gesundheitsförderung statt, beispielsweise in Form der kommunalen Gesundheitskonferenzen [13]. Hierzu gehört die wissenschaftliche Beforschung einer lebensweltorientierten und sektorenübergreifenden Gesundheitsplanung und Gesundheitsförderung im Umgang mit vulnerablen Individuen, sozialen Gruppen, Organisationen und Institutionen im digitalen Wandel.

### 8. Prävention inter- und transdisziplinär und auf unterschiedlichen Ebenen denken.

Prävention sollte über biopsychosoziale Ansätze, u. a. orientiert an der Internationalen Klassifikation der Funktionsfähigkeit, Behinderung und Gesundheit (ICF), so breit wie möglich abgebildet werden – hier ist eine möglichst ganzheitliche und umfassende Definition von „Gesundheit“ anzusetzen, und die Verschränkung des „Biopsychosozialen“ ist dementsprechend definitorischer Bestandteil der Wissenschaft von den Alternsprozessen [5]. Auch die Schutzfaktoren von Gesundheit und die Schutzreaktionen bei unterschiedlichen Stressoren sind biopsychosozial.

Im Bereich der Geriatrie ist die am ICF orientierte multiprofessionelle, funktionelle Rehabilitation in der Akutgeriatrie und der stationären und ambulanten geriatrischen Rehabilitation im Sinne der Prävention geeignet, Pflegebedürftigkeit zu verhindern/vermindern und die individuelle Selbstständigkeit mit verbesserter Lebensqualität bis ins hohe Alter zu erhalten.

Auf der psychischen Ebene können Resilienz im Umgang mit Belastungen sowie Selbstwirksamkeitserwartungen in Bezug auf die Aufnahme und Beibehaltung präventiven gesundheitsfördernden Verhaltens genannt werden [28]. Zur sozialen Ebene gehören soziale Determinanten von Gesundheit wie Unterstützungsnetzwerke und Austauschprozesse (inkl. Pflegearrangements und wohnortnaher geriatrischer Rehabilitation) in Gemeinschaften und Familien und die sozioökonomischen, gesellschaftlichen und umwelt-baulichen Rahmenbedingungen (inkl. technischer Unterstützung). Kontextuelle Ansätze schließen auf einer Makroebene regionale, politisch-historische und soziokulturelle Aspekte von Prävention und Gesundheitsförderung mit ein. Die digitale Transformation mit Möglichkeiten von Big Data, Wearables und Künstlicher Intelligenz ist untrennbar mit künftigen Präventionsmaßnahmen verwoben, wobei dennoch die *digital divide* minimiert werden muss [22].

Die Wechselwirkungen dieser biopsychosozialen Maßnahmen in einer inter- und transdisziplinären Präventionsforschung zu betrachten, kann deren Effektivität und Passung erhöhen [20]. Dabei sind etwa Prozesse der Zellalterung zentral, die mit immunologischen und entzündlichen Prozessen wechselwirken und z. B. durch richtige Bewegung und Ernährung aufgehalten werden können; dabei sind Biomarker wichtig, um diese Prozesse zu verstehen und die Wirkung von Interventionen vorherzusagen [17]. Es muss für eine umfassende, wirksame und lebensweltlich relevante Präventionsforschung und -praxis wesentlich stärker als in der Vergangenheit auf eine Integration der genannten Felder von molekular, zellulär, physiologisch, psychosozial, sozialräumlich und gesellschaftlich von der Theoriebildung bis hin zur Implementierung hingewirkt werden [3].

## Diskussion

Das vorliegende Papier zeigt mögliche Themen- und Handlungsfelder für politisch Entscheidungstragende und Praktiker:innen auf. Es richtet sich an die wichtigsten gesellschaftlichen Anspruchsgruppen, insbesondere an die (Fach)Öffentlichkeit, politische Entscheidungsträger sowie an die Allgemeinheit mit dem Aufruf, die Forschungslandschaft und -infrastruktur zur Prävention im Alter zu stärken und die Leitgedanken einer zukunftsgerichteten Alterns‑, Gesundheits- und Versorgungsforschung in Entwicklungen aufzunehmen.

Während das vorliegende Papier die Leitgedanken zur Stärkung der Prävention und Gesundheitsförderung im und für das Alter darlegt, benötigt es konkrete Umsetzungen und eine Akzentuierung präventiver Ansätze im Gesundheitswesen – in allen Settings und in Bezug auf eine Vielzahl an Erkrankungen – um diese Leitgedanken wirkungsvoll umzusetzen (konkrete Vorschläge dazu finden sich in der Online-Tabelle zu dieser Publikation). Der klare Fokus auf Stärkung von Entwicklungspotenzialen auch im höchsten Alter steht in keinem Widerspruch dazu, im Einzelfall auch Grenzen von Prävention anzuerkennen, denen häufig eine Kombination aus mangelnden Teilhabechancen, eingeschränkter biologischer Reservekapazität, internalisierten Altersbildern und -erwartungen sowie biografisch begründeten Präferenzen und Selbstwirksamkeitserwartungen zugrunde liegt.

Es bedarf eines Richtungswechsels hin zu kollaborativen, interprofessionell ausgerichteten und verhaltens- und verhältnisbezogenen (nichtpharmakologischen) Präventionsprogrammen für Menschen in der zweiten Lebenshälfte, die geeignet sind, die insbesondere für das sehr hohe Alter typische Multimorbidität, Frailty und Pflegebedürftigkeit zu vermeiden/vermindern und zu einer hohen Lebensqualität im Alter unabhängig vom sozioökonomischen Status beizutragen. Und obwohl es Beispiele für kollaborative Projekte, etwa aus der Versorgungsforschung (gefördert vom Gemeinsamen Bundesausschuss), gibt, fehlt es vielfach an der Implementierung (Zusatzmaterial online: Tabelle).

Diese interprofessionellen Präventionsprogramme sollten durch eine starke Evidenzgrundlage, die die Kontexte im deutschsprachigen Raum mitdenkt, getragen werden. Forschungsförderung sollte ferner der Untersuchung der Nachhaltigkeit von Effekten bzw. Langzeiteffekten stärker als bisher Rechnung tragen.

Schließlich ist die Implementierbarkeit von Präventionsmaßnahmen und Maßnahmen zur Gesundheitsförderung in das Gesundheitssystem wissenschaftlich zu prüfen, zu vereinfachen und auszubauen. Nicht nur einzelne Präventionsprojekte flächendeckend zu implementieren, sondern grundlegend die gesamte „Logik“ des Gesundheitssystems stärker auf die Prävention auszurichten, wäre ein fundamentaler, aber wesentlicher Schritt hin zu einer angemessenen Antwort auf die demografischen Herausforderungen unserer Zeit.

### Supplementary Information




