# Supplementary material for: Strengthening prevention and health promotion in and for old age
Source: Z Gerontol Geriatr. 2023 Dec 13;57(3):199–206. [Article in German] doi: 10.1007/s00391-023-02262-4 (PMC11078798; doi:10.1007/s00391-023-02262-4)
Supplement: Supplementary file 1 [file 391_2023_2262_MOESM1_ESM.pdf]

Appendix Tabelle 1. Themenfelder und Beispiele konkreter Handlungsempfehlungen

**Themenfeld**	**Beispiele konkreter Handlungsempfehlungen**
Prävention und Gesundheitsförderung im und für das Alter stärken	● Hier können übergeordnete Empfehlungen eingeordnet werden<br>● Edukative Ansätze für Prävention und Gesundheitsförderung an geragogischen Prinzipien (Prinzipien für Lernen im Alter) anlegen<br>● Begleitungansätze (gesundheitswissenschaftlich "Peer to Peer" Ansätze) zur Förderung von Gesundheitskompetenzen, zur Entscheidungshilfe, zur Nutzung von Angeboten als Ansatz stärken und fördern
1. Prävention und Gesundheitsförderung sind bis ins hohe Alter sinnvoll und möglich	● Alters-Cut-Offs für Studien überdenken, sowie generell die Bedeutung des rein chronologischen Alters überdenken, da es oft wenig aussagekräftig ist<br>● Angebote für das hohe Alter und an die Bedarfe (z.B. bezogen auf Zugang, Durchführung, Transfer) anpassen
2. Prävention und Gesundheitsförderung für das Alter sollten früh beginnen	● Gezielt strategische Konzepte entwickeln für frühzeitige auf das Alter gerichtete Prävention und Gesundheitsförderung - Altern als lebenslangen Prozess begreifen<br>● Wirksame, sichere und erschwingliche Interventionen im jungen und mittleren Lebensalter entwickeln/verbessern (Ernährung, Sport, …)
3. Prävention und Gesundheitsförderung müssen die Diversität und Heterogenität der Lebenslagen alter Menschen aufgreifen	● Die Reduktion von sozialer Ungleichheit mit als Endpunkt für Präventionsstudien verwenden<br>● Konzepte zu Beratung und Aufklärung inhaltlich, sprachlich und bezogen auf die vielfältigen Bedarfe resultierend aus den Lebenslagen anpassen
4. Prävention und Gesundheitsförderung fördern und fordern Selbstbestimmung und Partizipation	● Empowerment und Gesundheitskompetenz neben klinischen Endpunkten in Studien erheben<br>● Partizipative Forschung fördern
5. Prävention von Mehrfacherkrankungen gilt es stärker in den Blick zu nehmen	● Mehrfacherkrankungen, funktionaler Status und gesundheitsbezogene Lebensqualität als wichtige Endpunkte anerkennen<br>● Interventionen zur Prävention auf Multimorbidität statt ausschließlich auf Einzelerkrankungen ausrichten und fördern
6. Prävention von Pflegebedürftigkeit und Prävention in der Pflege sind gleichrangig zu behandeln	● Aufwertung nicht-pharmakologischer gegenüber pharmakologischen Interventionen, individuell passgenaue Förderung sinnvoller ernährungs- und bewegungsassoziierter Aktivitäten als "dauerhaft notwendiges Medikament ohne Zuzahlung"
7. Prävention und Gesundheitsförderung müssen lebensweltlich und sektorenübergreifend gedacht werden, dabei sind vor allem Aspekte der sozialen Ungleichheit und eine	● Kommunale Gestaltungsmöglichkeiten des Präventionsgesetzes unterstützen und ausschöpfen: Sektorenübergreifende Zusammenarbeit von Akteur:innen wie „stationäre und ambulante Versorgung", „Pflege", „Rehabilitation", „Prävention und Gesundheitsförderung" sowie „Stadtentwicklung", „Energie", „Verkehrsplanung" und „Digitalisierung".

Ressourcenorientierung mit zu beachten	• Gesundheitsförderung in den Lebenswelten älterer, alter und hochaltriger Menschen stärken und in die Lebenswelten integrieren<br>• Benennen und Auswählen, welche Lebenswelten hier einbezogen werden müssen und Veränderungen in den Lebenswelten in den Ansätzen mitdenken und Kontinuität bei Veränderungen gewährleisten<br>• Stärkung ressourcen- statt defizitorientierter Ansätze der Gesundheitsförderung
8. Prävention und Gesundheitsförderung und die Forschung dazu sind inter- und transdisziplinär und auf unterschiedlichen Ebenen anzulegen	• DFG: Eigenes Fach für Gerontologie und Alternsforschung einführen<br>• BMBF: Förderprogramme zum Thema Prävention und Gesundheitsförderung im Alter<br>• Helmholtz-Zentrum: stärker interdisziplinär und auf Prävention ausrichten<br>• G-BA Innovationsfonds: Ausschreibungen mit Themenschwerpunkt zu neuen Versorgungsformen und Versorgungsforschung im Bereich Prävention im und für das Alter<br>• Insgesamt die Ausrichtung auf Prävention im und für das höhere Lebensalter sowie auf die Kriterien Inter- und Transdisziplinarität in der Gesundheitsforschung bei der Mittelvergabe/ der Zuwendung von Forschungsgeldern stärken
